# Research Progress on Signaling Pathways in Breast Cancer Bone Metastasis

**DOI:** 10.32604/or.2026.079898

**Published:** 2026-09-14

**Authors:** Yunhong Li, Tianqi Li, Fenglian Liang, Lu Kuang, Yizhi Li, Hongsheng Li

**Affiliations:** 1 Department of Breast Surgery, Affiliated Cancer Hospital & Institute of Guangzhou Medical University, 78 Hengzhigang Rd, Guangzhou, China; 2 Southern Medical University Hospital of Integrated Traditional Chinese and Western Medicine, Southern Medical University, Guangzhou, China; 3 The Third Affiliated Hospital of Guangzhou Medical University, Guangzhou, China; 4 The Affiliated Guangzhou Twelfth People’s Hospital, Guangzhou Medical University, Guangzhou, China; 5 The Affiliated Cancer Hospital & Institute of Guangzhou Medical University, Guangzhou, China; 6 Department of Oncology, Tongji Hospital, Tongji Medical College, Huazhong University of Science and Technology, Wuhan, China

**Keywords:** Breast cancer (BC), bone metastasis, signaling pathway, bone microenvironment, targeted therapy

## Abstract

Breast cancer (BC) has become the most commonly diagnosed malignant tumor among women worldwide, with approximately 70% of patients with advanced BC developing bone metastases. These metastases trigger bone destruction and skeletal-related events (SREs) and significantly reduce patient survival. In recent years, research into the mechanisms underlying BC bone metastasis has advanced rapidly. Molecular biological and genomic studies have revealed that BC bone metastasis is co-regulated by multiple signaling pathways through crosstalk between BC cells and the bone microenvironment. This review analyzes the research progress of signaling pathways involved in BC bone metastasis and systematically elaborates four core cascades: Wingless-related integration site (Wnt)/β-catenin, transforming growth factor-β (TGF-β), RANK/receptor activator of nuclear factor-κB ligand (RANKL)/osteoclastogenesis inhibitory factor (OPG), and phosphatidylinositol 3-Kinase (PI3K)/protein kinase B (AKT)/mammalian target of rapamycin (mTOR). It explains how each pathway mediates epithelial-mesenchymal transition (EMT), excessive Osteoclast (OC) activation, maintenance of cancer stem cell stemness, and the formation of an immunosuppressive microenvironment. The positive feedback loops and reciprocal crosstalk between these pathways are also summarized, which together fuel the vicious cycle of osteolytic bone metastasis. This paper further consolidates therapeutic strategies targeting the aforementioned signaling pathways and outlines cutting-edge therapeutic approaches and emerging research hotspots. Nevertheless, critical obstacles including complex pathway compensation, drug resistance, and dysregulated bone immunity remain major bottlenecks hindering clinical translation. Future research will leverage single-cell sequencing and multi-omics technologies to identify pivotal molecular targets and develop potent combinatorial therapies. Such advances will facilitate the implementation of precise, individualized treatment for BC bone metastasis and ultimately improve the quality of life and long-term clinical outcomes of patients with advanced bone-metastatic disease.

## 1 Introduction

According to the latest data released by the International Agency for Research on Cancer (IARC) of the World Health Organization in 2022, BC has become the most common malignant tumor among women globally, with approximately 2.3 million new cases worldwide, accounting for 11.6% of all new cancer cases globally. It ranks second in the global cancer incidence spectrum, following lung cancer [1], and causes 685,000 deaths, representing 24.5% of female cancer incidence and 15.5% of cancer-related deaths [2], imposing a heavy burden on patients, their families, and society. The metastatic routes of BC include local invasion, lymphatic metastasis, and hematogenous metastasis, among which lymphatic and hematogenous spread are the most common. In hematogenous metastasis of BC, bone, brain, and liver are frequent distant metastatic sites. Bone metastasis is a common complication in advanced BC and a major cause of death; it is the third most common metastatic site in BC, with up to 70% of patients with metastatic BC presenting with bone involvement [3]. The mechanisms underlying BC bone metastasis involve a multifactorial, multi-step complex process, encompassing cancer cell detachment, circulation, and subsequent colonization, proliferation, and bone destruction within the bone microenvironment, which involves epithelial–mesenchymal transition (EMT), activation of matrix metalloproteinases (MMPs), and the influence of the tumor microenvironment. A summary of the acronyms and abbreviations mentioned throughout the manuscript are summarized in Table 1.

**Table 1:** Acronym Summary Table.

Category	Abbreviation	Full Name
Institution	IARC	International Agency for Research on Cancer
Cell Biology Processes	EMT	Epithelial-mesenchymal Transition
Survival & Clinical Endpoint	DMFS	Distant Metastasis-free Survival
	DFS	Disease-free Survival
	PFS	Progression-free Survival
	OS	Overall Survival
	CBR	Clinical Benefit Rate
	ORR	Objective Response Rate
	SRE	Skeletal-Related Event
Cell Type (Osteocyte)	OB	Osteoblast
	OC	Osteoclast
Cell Type (Immunocyte)	DC	Dendritic Cell
	Treg	Regulatory T Cell
	CTL/CD8^+^ T	Cytotoxic T Lymphocyte
	NK	Natural Killer cell
	TAM	Tumor-Associated Macrophage
Cell Type (Cancer Cell)	BCSC	Breast Cancer Stem Cell
	CTC	Circulating Tumor Cell
	CAF	Cancer-associated Fibroblasts
Cytokine & Growth Factor	TGF	Transforming Growth Factor
	Pro-TGF-β	Transforming Growth Factor-β Proprotein
	BMP	Bone Morphogenetic Protein
	VEGF	Vascular Endothelial Growth Factor
	EGF	Epidermal Growth Factor
	FGF	Fibroblast Growth Factor
	PDGF	Platelet-derived Growth Factor
	IGF	Insulin-like Growth Factor
	CTGF	Connective Tissue Growth Factor
	M-CSF	Macrophage Colony-stimulating Factor
	IL	Interleukin
	TNF	Tumor Necrosis Factor
	RANKL	Receptor Activator of Nuclear Factor-κB Ligand
	mRANKL	Membrane-bound RANKL
	sRANKL	Soluble RANKL
	PG	Prostaglandin
	PTHrP	Parathyroid Hormone-related Protein
	OPG	Osteoclastogenesis Inhibitory Factor
	SOST	Sclerostin
Transcription Factor	TF	Transcription Factor
	Sp1	Specificity Protein 1
	Runx	Runt-related Transcription Factor
	NFATc1	Nuclear Factor of Activated T-cells cytoplasmic 1
	FOXO	Forkhead Box O
	HIF	Hypoxia-inducible Factor
	IGF-1R	Insulin-like Growth Factor 1
	Osx/SP7	Osterix
	SGK1	Serum and Glucocorticoid-regulated Kinase
Signaling Pathway Molecules (Wnt/β-catenin)	Wnt	Wingless-related Integration Site
	WTX	Wilms Tumor on The X Chromosome
	WLS	Wntless
	Fz	Frizzled
	LRP	LDL Receptor-related Protein
	Dvl	Dishevelled
	APC	Adenomatous Polyposis Coli
	GSK	Glycogen Synthase Kinase
	β-TrCP	β-transducin Repeat-containing Protein
	TCF	T-cell factor
	LEF	Lymphoid Enhancer Factor
	Pygo	Pygopus
	BCL9	B-cell CLL/lymphoma 9
	Brg1	Brahma-related gene 1
	CBP	cAMP Response Element Bound Protein Binding Protein
	WIF	Wnt Inhibitory Factor
	SFRP	Secreted Frizzled-related Protein
	DKK	Dickkopf-related Protein
	ICAT	Inhibitor of β-catenin and TCF
	TAZ	Transcriptional Coactivator with PDZ-binding Motif
Signaling Pathway Molecules (TGF-β)	SLC	Small Latent Complex
	LTBP	Latent TGF-β Binding Protein
	LLC	Large Latent Complex
	R-Smad	Receptor-regulated Smad Protein
	Co-Smad	Common-type Smad Protein
	I-Smad	Inhibitory Smad Protein
	LAP	Latency-associated Peptide
Signaling Pathway Molecules (PI3K/AKT/mTOR)	PI3K	Phosphatidylinositol 3-kinase
	P13KIA	Class IA Phosphoinositide 3-kinase
	AKT/PKB	Protein Kinase B
	mTOR	Mammalian Target of Rapamycin
	PIP2	Phosphatidylinositol 4,5-bisphosphate
	PIP3	Phosphatidylinositol 3,4,5-trisphosphate
	TSC	Tuberous Sclerosis Complex
	PTEN	Phosphatase and Tensin Homolog
	PRAS40	Proline-rich AKT Substrate of 40 kDa
	4E-BP1	eIF4E-binding Protein
	S6K1	Ribosomal Protein S6 Kinase Beta-1
	Rheb-GTP	Ras Homolog Enriched in Brain-GTP
	ATP	Adenosine Triphosphate
	RAS	Rat Sarcoma Virus
Signaling Pathway Molecules (Other)	MAPK	Mitogen-activated Protein Kinase
	FAK	Focal Adhesion Kinase
	CDK	Cyclin-dependent Kinase
	ERK	Extracellular Signal-regulated Kinase
	CK1	Casein Kinase 1
	JNK	Jun N-terminal kinase
	PDK1	3-Phosphoinositide-dependent Protein Kinase 1
	SRC	Non-Receptor Tyrosine Kinase
	PIKK	Phosphatidylinositol Kinase-related Kinase
	SGK	Serum and Glucocorticoid-regulated Kinase
	PK	Protein Kinase
	NLK	Nemo-like Kinase
	RTK	Receptor Tyrosine Kinase
	CN	Calcineurin
	GLI2	GLI Family Zinc Finger 2
	TRAF	TNF Receptor-associated Factor
	GPCR	G Protein-coupled Receptor
Enzyme & Matrix Protein	HDAC	Histone Deacetylase
	CR	Chromatin Remodeler
	MMP	Matrix Metalloproteinase
	ECM	Extracellular Matrix
	CTSK	Cathepsin K
	TRAP	Tartrate Resistant Acid Phosphatase
	BSP	Bone Sialoprotein
	BAD	B-cell Lymphoma-2 Gene-related Promoter
	ANXA2	Annexin A2
	CYLD	CYLD Lysine 63 Deubiquitinase
Receptors, Domain & Protein Modifications	PTH1R	PTH1 Receptor
	ERα	Estrogen Receptor α
	TβR	TGF-β Type Receptor
	IRS-1	Insulin Receptor Substrate 1
	IGF-1R	Insulin-like Growth Factor 1 Receptor
	CRD	Cysteine-rich Domain
	SH2	Src Homology 2
	RNF	Ring Finger Protein
	ZNRF	Zinc and Ring Finger
	LGR5/6	Leucine-rich Repeat-containing G Protein-coupled Receptor 5/6
	CXCR4/CD184	C-X-C Motif Chemokine Receptor 4

## 2 Background of BC Bone Metastasis

In 1889, Stephen Paget first described the complexity of bone metastasis, proposing the “seed and soil” hypothesis to explain the tendency of tumor cells to metastasize to certain organs. When tumor cells mature (like plant seeding), the seeds are carried to various parts of the body but only grow in suitable organs (the appropriate soil), eventually forming metastatic lesions. In BC bone metastasis, BC cells exhibit high tropism for bone, activating OCs to degrade the bone matrix, thereby creating a microenvironment favorable for tumor cell colonization [4,5].

BC bone metastases frequently occur in bones rich in red bone marrow and trabecular bone, such as the thoracic and lumbar vertebrae, ribs, pelvis, and the ends of long bones, while metastases are rarely observed in the bones of the extremities [6]. This process involves complex interactions among multiple cell types and signaling pathways. Once BC metastasizes to bone, it significantly increases patient mortality and treatment difficulty, leading to bone-related complications including severe pain, increased fracture risk, nerve compression, hypercalcemia, and bone marrow suppression, which collectively contribute to a poor clinical prognosis [7]. Bone metastases are generally classified into osteoblastic (commonly seen in prostate cancer) and osteolytic (commonly seen in BC) types [8,9], with osteolytic, mixed, and osteoblastic metastases accounting for 48%, 38%, and 13% of cases, respectively [7]. Approximately 70% of patients with advanced BC develop bone metastases, with a median survival of 13–47 months following bone metastasis, varying by age, subtype, and the presence or absence of visceral metastase [10]. In osteolytic lesions, bone resorption is increased but compensatory bone formation is impaired; in osteoblastic lesions, bone formation is disorganized and bone resorption is defective.

BC bone metastasis is a multi-step, multifactorial biological process involving local invasion of BC cells, detachment from the primary tumor, survival in the circulation, homing to the bone marrow, colonization within the bone microenvironment, and a vicious cycle of osteolytic and osteoblastic activity. Tumor cells, osteoblasts (OBs), osteoclasts (OCs), and bone matrix constitute the four essential components of this vicious cycle required for the initiation and progression of bone metastasis. This process is regulated by multiple molecular mechanisms, including remodeling of the extracellular matrix (ECM), changes in cell adhesion molecules, release of growth factors and cytokines, and activation of signaling pathways. These signaling pathways not only influence the proliferation, migration, invasion, metastasis, and survival of BC cells but are also closely associated with the crosstalk between BC cells and the bone microenvironment, collectively promoting the occurrence and progression of BC bone metastasis. This article will discuss the major signaling pathways currently implicated in BC bone metastasis [5,7,11].

## 3 The Synergistic Roles of the Four Classic Signaling Pathways in BC Bone Metastasis

BC bone metastasis is a major cause of death, involving interactions among multiple cell types and signaling pathways [12]. This review focuses on the roles of four major signaling pathways—Wnt/β-catenin, TGF-β, RANK/RANKL/OPG, and PI3K/AKT/mTOR—in BC bone metastasis and discusses how they affect tumor cell proliferation, migration, invasion, metastasis, survival, and their crosstalk with the bone microenvironment.

### 3.1 The Wnt/β-Catenin Signaling Pathway

#### 3.1.1 Basic Mechanisms of the Wnt/β-Catenin Signaling Pathway

Currently, 19 Wnt proteins have been identified in humans, all of which are secreted glycoproteins. The Wnt signaling pathway is mainly divided into the canonical Wnt/β-catenin-dependent pathway and non-canonical pathways (including the Wnt/Ca^2+^ pathway and the Wnt/PCP pathway). The canonical Wnt/β-catenin signaling pathway is a highly conserved and complex mechanism that exerts profound effects on embryonic development, tissue homeostasis, and tumor metastasis. Non-canonical Wnt pathways do not depend on β-catenin; instead, they regulate the cytoskeleton, cell polarity, and directional migration, and are essential for tissue morphogenesis during embryonic development [13].

In the absence of Wnt ligand stimulation, the pathway remains in a silent state, characterized by continuous degradation of β-catenin in the cytoplasm to maintain a low steady-state level of this protein within the cell. This process is precisely regulated by a “degradation complex” composed of Axin, adenomatous polyposis coli (APC) protein, glycogen synthase kinase 3α/β (GSK-3α/β), and casein kinase 1 (CK1), accompanied by transcriptional repression of downstream target gene [14]. Cytoplasmic β-catenin functions primarily through two mechanisms. First, β-catenin interacts with E-cadherin, α-catenin, and p120 at the cell membrane, which helps establish and stabilize cell–cell adhesion junctions, thereby playing a critical role in maintaining the structural integrity of epithelial tissues [15]. Second, free cytoplasmic β-catenin serves as a target for the degradation complex. Tankyrase indirectly affects the stability of the β-catenin degradation complex by ribosylating and degrading Axin [16]. PP2A stabilizes the structure of the degradation complex through dephosphorylation of Axin [15].

**Figure 1:**
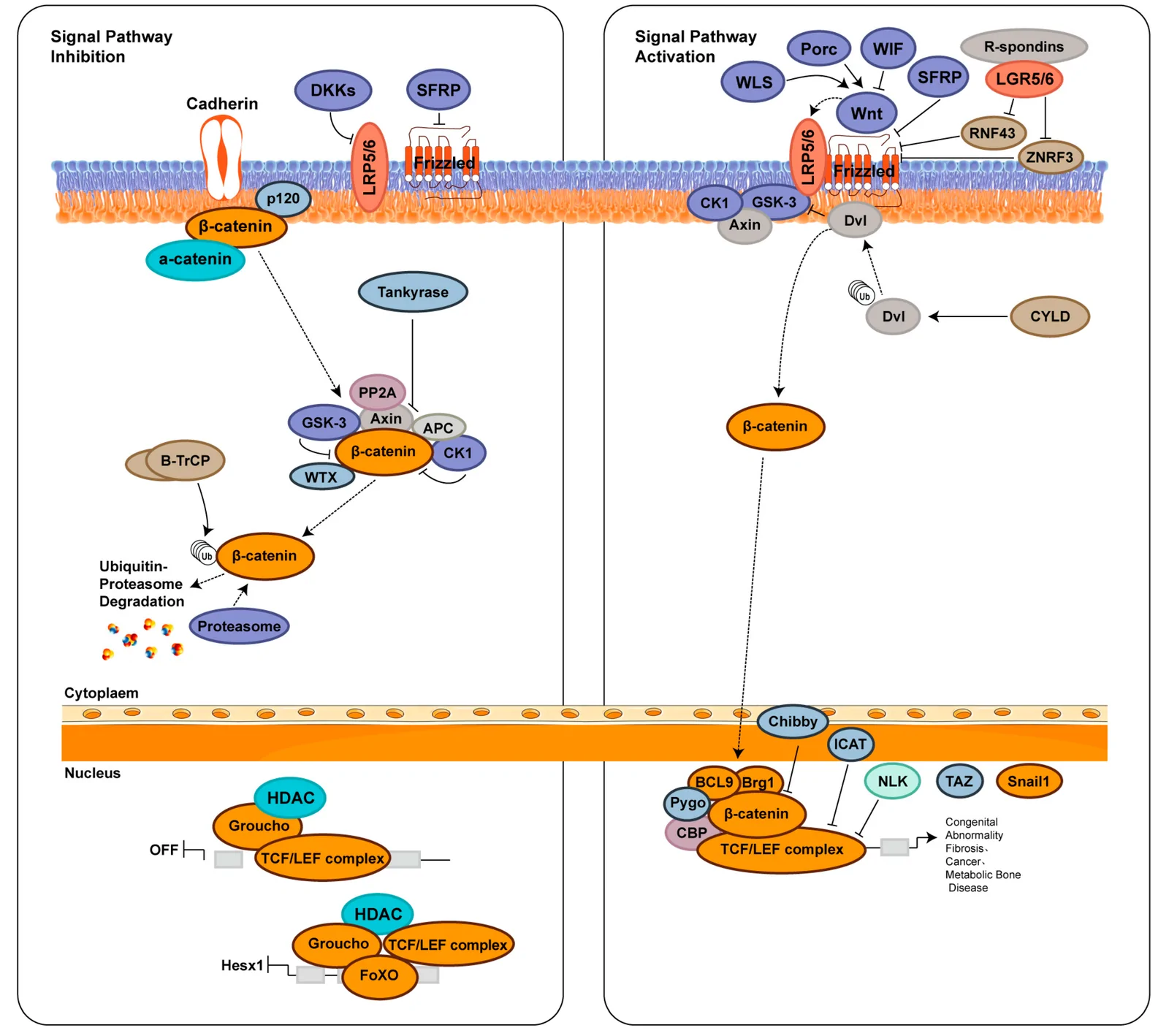
Schematic diagram of the canonical Wnt/β-catenin signaling pathway. The left side shows the pathway in the ‘off’ state: inhibitory factors such as the DKK and SFRP families, together with the degradation complex, promote β-catenin degradation, thereby blocking target gene expression. The right side shows the pathway in the activated state: upon binding of Wnt ligands to receptors, the function of the degradation complex is inhibited, allowing β-catenin to accumulate in the cytoplasm and translocate into the nucleus, where it binds to the TCF/LEF transcriptional complex and initiates the transcription program of target genes. Figure generated with Adobe Illustrator 2026 (Adobe, USA).

When the Wnt/β-catenin signaling pathway is in its resting (off) state, β-catenin is continuously degraded through a phosphorylation–ubiquitination–proteasome degradation cascade, maintaining a low intracellular concentration. Specifically, this process is mediated by the β-catenin destruction complex composed of Axin, APC, CK1 and GSK-3β in the cytoplasm. CK1 first primes β-catenin by phosphorylating its Ser45 residue, which lays the groundwork for subsequent modification by GSK-3β. GSK-3β then sequentially phosphorylates Thr41, Ser37 and Ser33 of β-catenin to generate a characteristic phosphorylated motif. The phosphorylated β-catenin is recognized by β-TrCP for ubiquitination and is eventually degraded via the 26S proteasome. This maintains β-catenin at a low nanomolar concentration within the cytoplasm, blocks its nuclear translocation, and thereby suppresses the transcription of target genes. Target gene silencing in the pathway-off state is not solely dependent on the absence of β-catenin, but is also actively regulated by transcriptional repressor complexes. In the nucleus, T-cell factor (TCF)/lymphoid enhancer factor (LEF) family transcription factors bind to conserved sequences in the promoters of target genes, while simultaneously associating with Groucho family co-repressors, which recruit histone deacetylases (HDACs) to reduce chromatin transcriptional activity. In addition, proteins such as forkhead box O1 (FOXO1) and Hesx1 can directly bind to target gene promoters or interact with the TCF/LEF transcriptional complex, further reinforcing transcriptional silencing [15,17] (Fig. 1 left panel).

Upon the binding of Wnt ligands to cell membrane receptors, the Wnt/β-catenin signaling pathway is activated and β-catenin degradation is blocked. Wnt ligands are assembled and matured via Porc and wntless (WLS) proteins, transported through the endoplasmic reticulum, and secreted extracellularly [18]. They then bind to frizzled (Fz) receptors in an autocrine/paracrine manner, recruiting LDL receptor-related protein5 and 6 (LRP5/6) to assemble a Frizzled-LRP5/6-Wnt ternary signaling complex. The core of signal activation is the sequential phosphorylation of the intracellular domain of LRP5/6: CK1 first phosphorylates Ser1490, followed by GSK-3 phosphorylation of multiple adjacent serine residues, forming a phosphorylation cluster that promotes the recruitment of Axin to the cell membrane, directly leading to the disassembly of the cytoplasmic β-catenin degradation complex. Simultaneously, dishevelled (Dvl) proteins bind to Fz receptors and undergo oligomerization, which inhibits GSK-3 activity, further blocking β-catenin phosphorylation and degradation [19]. Following degradation complex disassembly, unphosphorylated β-catenin rapidly accumulates in the cytoplasm and translocates into the nucleus. Nuclear β-catenin competitively displaces Groucho repressors bound to the TCF/LEF transcriptional complex via its N-terminal domain and subsequently recruits co-activators such as B-cell CLL/lymphoma 9 (BCL9), pygopus (Pygo), brahma-related gene 1 (Brg1), and cAMP response element bound protein binding protein (CBP)/p300 to assemble a transcriptional activation complex. Among these, Pygo is responsible for localizing the complex to chromatin, Brg1 remodels nucleosome structure and enhances promoter accessibility, and CBP/p300 integrates signals from multiple pathways, ultimately coordinating the transcription of Wnt downstream target genes (Fig. 1 right panel).

The Wnt/β-catenin pathway is subject to multiple layers of fine-tuned negative feedback inhibition to prevent excessive pathway activation. At the extracellular level, secreted frizzled-related proteins (SFRPs) contain an N-terminal cysteine-rich domain (CRD) homologous to the extracellular region of Fz receptors. Via this domain, SFRPs either compete with Fz receptors for binding to Wnt ligands or directly form non-functional complexes with Fz receptors, thereby hindering the interaction between Wnt ligands and Fz receptors. Wnt inhibitory factors (WIFs) bind to Wnt ligands with high affinity, forming stable complexes that prevent ligand-mediated signal activation. At the receptor level, dickkopf-related protein (DKK) acts as a specific, high-affinity antagonist of LRP5/6, occluding its ligand-binding sites and inhibiting the assembly of the Frizzled-LRP5/6-Wnt ternary complex. Simultaneously, overactivation of the Wnt pathway upregulates the expression of zinc and ring finger 3 (ZNRF3)/ring finger protein 43 (RNF43), which recognize and bind to Wnt receptors, mediating their ubiquitination and delivery to lysosomes for degradation, thereby downregulating pathway activity and forming a classic negative feedback loop. Within the nucleus, multiple inhibitory safeguards also exist, with inhibitor of β-catenin and TCF (ICAT), Chibby, and nemo-like kinase (NLK) serving as core nuclear inhibitors: ICAT blocks the binding of β-catenin to TCF; Chibby competitively inhibits the recruitment of CBP/p300 by β-catenin; and NLK reduces the binding affinity between TCF and β-catenin through phosphorylation. These three proteins act synergistically to suppress downstream Wnt/β-catenin signaling, maintaining cellular homeostasis and preventing oncogenesis. Conversely, R-spondins exert positive regulatory effects by binding to LRP5/6 receptors, further recruiting ZNRF3/RNF43 for degradation; this relieves the ubiquitination of Wnt membrane receptors, stabilizing Fz and LRP5/6 levels on the cell surface and rendering cells highly sensitive to minute amounts of Wnt ligands, thus forming a positive feedback mechanism [20,21,22].

#### 3.1.2 The Role of the Wnt/β-Catenin Signaling Pathway in BC Bone Metastasis

##### Regulation of Bone Homeostasis by the Wnt/β-Catenin Pathway

The Wnt family is a candidate factor family associated with OBs, with Wnt1, Wnt3A, and Wnt5A playing important roles in the regulation of skeletal development and bone formation. Under normal conditions, activation of the Wnt/β-catenin pathway promotes osteogenic differentiation, bone matrix synthesis, and upregulation of OPG. Meanwhile, Wnt ligands and other cytokines [such as Bone morphogenetic protein (BMPs), OPG, etc.] secreted by OBs in a paracrine manner act on OCs, inhibiting their differentiation and activity, thereby reducing bone resorption [23,24].

##### Wnt/β-Catenin Pathway Facilitates Migration, Invasion and Bone Homing of BC Cells

Aberrant activation of the Wnt/β-catenin pathway serves as a core driving force in BC bone metastasis, mediating the migration of BC cells from the primary tumor, bone colonization, and sustained osteolytic destruction. Within BC cells, ligands such as Wnt1 and Wnt3A are highly expressed and bind to the FZD/LRP5/6 co-receptors on the cell membrane, inhibiting the key negative regulator GSK-3β. This prevents the ubiquitination and degradation of β-catenin, allowing its accumulation in the cytoplasm, followed by nuclear translocation and the formation of a transcriptional complex with TCF/LEF, which activates downstream metastasis-related target genes. This pathway directly upregulates transcription factors such as ZEB1, Snail, and Twist, driving EMT, while also inducing the expression of MMP-2/MMP-9, which degrade the ECM and basement membrane, significantly enhancing the migration and invasion abilities of BC cells. Furthermore, the Wnt/β-catenin pathway activates bone-directed metastasis mediators such as HOXB9 and LEF-1, upregulates the C-X-C motif chemokine receptor 4 (CXCR4), and enhances the chemotactic response of BC cells to CXCL12, which is highly expressed in bone tissue, thereby achieving efficient bone homing and colonization [20,25].

##### Wnt/β-Catenin Pathway Mediates the Formation of Osteolytic Bone Microenvironment and Positive Feedback Loop

Bone microenvironment remodeling is a core step in BC bone metastasis driven by the Wnt/β-catenin pathway, which establishes a vicious cycle of OBs inhibition and OCs activation, creating an osteolytic microenvironment conducive to tumor growth. Nuclear translocation of β-catenin in BC cells directly promotes the secretion of Wnt pathway inhibitors such as DKK-1 and sclerostin (SOST), which inhibit Wnt signaling in OBs via paracrine signaling, leading to suppressed OB differentiation and function and reduced bone formation. Simultaneously, decreased osteoprotegerin (OPG) secretion and elevated RANKL expression markedly increase the RANKL/OPG ratio, which promotes the binding of RANKL to the RANK receptor on OC surfaces, activating key osteoclastogenic transcription factors such as nuclear factor of activated T-cells cytoplasmic 1 (NFATc1) and c-Fos, inducing excessive OC proliferation, extensive bone matrix degradation, and the release of growth factors including TGF-β, BMPs, and IGF-1. These growth factors further activate the Wnt/β-catenin pathway in a paracrine manner, promoting the release of more osteolytic factors and forming a positive feedback loop that sustains the continuous growth of bone metastases and osteolytic bone destruction [25]. In addition, the Wnt/β-catenin pathway regulates tumor stemness by upregulating markers such as SOX2, OCT4, and Nanog, maintaining BC stem cell (BCSC) characteristics and enhancing their drug resistance, anti-apoptotic capacity, and long-term survival within the bone microenvironment [26].

##### Insights from *In Vitro* Models: Complex Effects of Wnt Signaling in BC Bone Metastasis

Sumanta Kar et al. used nanoclay scaffolds to establish a 3D *in vitro* BC bone metastasis microenvironment, finding that the Wnt/β-catenin pathway has a significant effect on osteogenesis during BC bone metastasis [27]. Research by Professor Liu’s group demonstrated that overexpression of Lrp5, a component of the Wnt signaling pathway, enhances the tumor-suppressive and bone-protective effects of osteoblast-conditioned medium, revealing the complex role of the Wnt signaling pathway in BC bone metastasis [28].

#### 3.1.3 Therapeutic Strategies Targeting the Wnt/β-Catenin Pathway for BC Bone Metastasis

Given the important role of the Wnt/β-catenin pathway in BC bone metastasis, targeting this pathway represents a novel and highly promising therapeutic direction for intervention. Current therapeutic approaches primarily focus on Wnt ligand or receptor antagonists, β-catenin degradation promoters, nuclear transcriptional complex inhibitors, bone microenvironment modulators, natural bioactive small-molecule drugs, and gene-targeted interventions. These strategies aim to inhibit aberrant activation of the Wnt/β-catenin pathway, restore bone microenvironment homeostasis, and suppress tumor cell proliferation and invasion, thereby delaying or preventing the occurrence and progression of BC bone metastasis [29].

##### Upstream Therapeutic Agents: Wnt Ligands and Receptor Antagonists

In terms of upstream targeting interventions of the pathway, the anti-Frizzled monoclonal antibody Vantictumab (OMP-18R5) specifically binds to FZD1/2/5/7/8 receptors, blocking the binding of Wnt ligands to receptors and inhibiting aberrant activation of the Wnt/β-catenin pathway. It effectively reduces BC stem cell activity and reverses EMT, thereby suppressing BC cell bone colonization. It has entered a Phase Ib clinical study in HER2-negative metastatic BC, and in combination with paclitaxel, it exerts synergistic anti-tumor effects and reduces the degree of bone destruction [30,31]. The Wnt ligand antagonist Ipafricept neutralizes key activating ligands such as Wnt3A and Wnt5A, reducing intracellular β-catenin activity. It has been shown in Phase I clinical studies to effectively inhibit OC differentiation and alleviate BC-mediated osteolytic bone destruction [32].

##### Core Pathway Inhibitors: Regulators of β-Catenin Degradation and Transcriptional Complex

Targeted intervention in the Wnt/β-catenin pathway primarily focuses on regulating β-catenin degradation and nuclear transcription processes, fundamentally blocking aberrant pathway activation. The GSK-3β inhibitor 1-azakenpaullone, by inhibiting GSK-3β activity, stabilizes β-catenin and specifically activates the Wnt pathway in OBs, promoting OPG secretion and inhibiting osteolysis [33]; inhibition of GSK-3β can also suppress EMT and BC stem cell properties [34]. In addition, the tankyrase inhibitor XAV939 [35,36] and the axin stabilizer IWR-1 [37] enhance the stability of the degradation complex and promote ubiquitination and degradation of β-catenin, offering new directions for foundational targeted therapy. Nuclear transcriptional complex inhibitors can precisely block downstream transcriptional activation; among these, ICG-001 inhibits the formation of the β-catenin/CBP transcriptional activation complex and downregulates the expression of c-Myc, Cyclin D1, and EMT-related metastatic genes [38]. PKF118-310 [39] interferes with the interaction between β-catenin and TCF, and CCT036477 [40] disrupts the binding of β-catenin to TCF, thereby blocking downstream metastasis-related signaling.

##### Therapeutic Agents Targeting Bone Microenvironment

In terms of bone microenvironment modulation, targeted intervention can effectively reverse the bone metabolic imbalance induced by BC bone metastasis, serving as an important strategy to ameliorate osteolytic bone destruction. The DKK-1 neutralizing antibody BHQ880 specifically antagonizes the Wnt pathway inhibitor DKK-1, which is highly expressed in the bone microenvironment, relieving its blockade of LRP5/6 receptors on OB membranes and reactivating the Wnt/β-catenin pathway in OBs, thereby promoting OB differentiation and trabecular bone formation while inhibiting OC activation and bone matrix dissolution [41]. Serum DKK1 levels are significantly elevated in BC patients with bone metastases, which in turn suppresses OB activity around metastatic lesions and exacerbates bone matrix dissolution. This drug effectively increases bone mineral density and reduces the incidence of SREs in patients [42,43]. In addition, romosozumab is a humanized anti-SOST monoclonal antibody (Scl-Ab) targeting SOST secreted by osteocytes. It blocks the binding of SOST to LRP5/6 on the OB membrane, relieving SOST-mediated inhibition of the Wnt pathway. This persistently stimulates OBs to synthesize bone matrix and facilitates bone formation, while suppressing osteoclast differentiation and bone resorption. In a mouse model of breast cancer tibial metastasis, romosozumab reduces the area of osteolytic lesions and decreases bone metastatic tumor burden. Meanwhile, it ameliorates tumor-associated muscular atrophy and prolongs the survival of tumor-bearing mice. Owing to its unique dual regulatory effects on bone metabolism, romosozumab has been approved by the FDA for the treatment of postmenopausal osteoporosis with high fracture risk, and is regarded as a promising bone-modifying agent for breast cancer bone metastasis. Nevertheless, phase III clinical studies investigating its efficacy in patients with breast cancer bone metastasis are still lacking [41,44].

##### Natural Small Molecules and Gene-Targeted Interventions

Natural bioactive small-molecule drugs [45] and gene-targeted interventions offer new approaches for the treatment of BC bone metastasis. Natural compounds such as curcumin [39], quercetin [46,47], resveratrol [48], and sulforaphane [49] can inhibit the Wnt/β-catenin pathway and possess combined anti-tumor, bone microenvironment-modulating, and anti-inflammatory/attenuating effects. They have been used for skin protection during radiotherapy and chemotherapy in BC, but have not yet been approved for the treatment of BC bone metastasis. Gene intervention technologies enable precise targeted regulation: siRNA and shRNA targeting the CTNNB1 gene can silence β-catenin expression, suppressing tumor proliferation and metastasis at the genetic level and prolonging the survival of tumor-bearing mice [50,51]. Meanwhile, siRNA targeting SOST can relieve the inhibited state of the Wnt pathway in the bone microenvironment, restore normal bone metabolic balance, and exert synergistic therapeutic effects when combined with anti-resorptive drugs [52,53,54]. The treatment regimens targeting the Wnt/β-catenin signaling pathway of breast cancer bone metastasis are listed in Table 2.

**Table 2:** Treatment Methods for the Four Major Signaling Pathways of Bone Metastasis in Breast Cancer.

Signaling Pathway	Drugs/Intervention Strategies	Type	Research Status	Function
Wnt/β-catenin	Vantictumab (OMP-18R5)	Anti-Frizzled monoclonal antibody	HER2-negative metastatic breast cancer Phase Ib clinical trial.	Inhibits tumor stem cell activity, reverses EMT, and, when used with paclitaxel, reduces mild bone damage.
	Ipafricept (OMP-54F28)	Wnt ligand antagonist	Phase I clinical trials for solid tumors.	Neutralizing Wnt3A, Wnt5A, etc., inhibits osteoclast differentiation and eases bone destruction.
	1-Azakenpaullone	GSK-3β inhibitor	Preclinical research	Inhibits GSK-3β activity, stabilizes β-catenin, specifically activates the Wnt pathway in osteoblasts, promotes OPG secretion, and suppresses bone resorption.
	XAV939	Tankyrase inhibitor	Preclinical research	Inhibit TNKS1/2 glycosylation modification of Axin, promote β-catenin degradation, and inhibit abnormal activation of the Wnt pathway.
	IWR-1	Axin stabilizer	Preclinical research	Change the conformation of the Axin protein to prevent TNKS degradation, promote β-catenin degradation, and inhibit abnormal activation of the Wnt pathway.
	ICG-001	Nuclear transcription complex inhibitor	Preclinical research	Competitively blocking β-catenin from binding to CBP, shutting down downstream target gene transcription, and inhibiting EMT and metastasis.
	PKF118-310	Nuclear transcription complex inhibitor	Preclinical research	Blocking the binding of β-catenin to TCF, stopping downstream target gene transcription, and inhibiting cancer stem cells and metastasis.
	CCT036477	Nuclear transcription complex inhibitor	Preclinical research	Same as above.
	BHQ880	Anti-DKK-1 antibody	Preclinical research	Relieve DKK-1’s suppression of Wnt signaling in osteoblasts, promote bone formation, and increase bone density.
	Scl-Ab/Romosozumab	Anti-SOST antibody	Already approved for osteoporosis, but lacks phase III clinical trial data for breast cancer bone metastases.	It relieves SOST’s inhibition of Wnt, regulates bone metabolism bidirectionally, promotes bone formation, inhibits bone resorption, and reduces bone lesions.
	Curcumin, quercetin, resveratrol, sulforaphane	Natural active small-molecule drug	Preclinical research	Block the Wnt/β-catenin pathway.
TGF-β	1D11	Mouse-derived ligand neutralizing antibody	Preclinical research	Lower RANKL expression and increase OPG.
	Fresolimumab (GC1008)	First-generation humanized ligand-neutralizing antibody	Phase II Clinical Study of Advanced Triple-Negative Breast Cancer.	It lowers TGF-β levels in peripheral blood, but its effect as a single drug is limited, and there are skin and cardiovascular side effects.
	SAR439459	Second-generation humanized ligand-neutralizing antibody	Only a few mixed cases, no dedicated cohort.	The plasma TGF-β inhibition rate is higher, reshaping the bone immune microenvironment.
	D10-Fc-T2m	Receptor Fc fusion protein	Preclinical research	Specifically accumulates in bone lesions and binds to, neutralizes TGF-β.
	Galunisertib (LY2157299)	Dual-target ALK5 inhibitor	Phase II clinical trials for advanced solid tumors have been completed, but there is a lack of Phase III clinical research for breast cancer bone metastasis.	At the same time, inhibit TGF-βRI/II, block the Smad pathway, and weaken bone resorption.
	Bicyclic phage display peptides	Smurf ubiquitin ligase inhibitor	Preclinical research	Blocking the binding of Smurf2 and Smad7, reducing Smad7 degradation, and inhibiting TGF-β induced EMT and PTHrP
RANK/RANKL/OPG	Denosumab (AMG162)	Fully human monoclonal antibody targeting RANKL	Already approved for first-line treatment of breast cancer with bone metastases.	It specifically neutralizes RANKL, inhibits osteoclast activation, reduces the risk of SREs, and doesn’t get metabolized by the kidneys.
	Zoledronic Acid	Third-generation bisphosphonates	Already approved as an alternative treatment for breast cancer bone metastasis.	Induces osteoclast apoptosis, indirectly downregulates RANK activity, is cleared through the kidneys, and is used for patients with normal kidney function.
	OPG mutant/LGR4-ECD	New type of decoy receptor	Preclinical research	Competing to bind RANKL is the direction for developing the next-generation single drug.
PI3K/AKT/mTOR	Alpelisib	Selective PI3Kα inhibitor	Approved for use in HR+/HER2− advanced or metastatic breast cancer carrying PIK3CA mutations.	When used with fulvestrant, it inhibits PTHrP and IL-6 secretion and extends overall survival.
	Inavolisib	Selective PI3Kα inhibitor	Approved for use in HR+/HER2− advanced or metastatic breast cancer carrying PIK3CA mutations.	When combined with palbociclib/fulvestrant, it shows a good overall survival benefit.
	Capivasertib	ATP competitive AKT inhibitor	Approved for treating HR+/HER2− advanced breast cancer patients who have relapsed or progressed during or after aromatase inhibitor therapy	Blocking the PI3K/AKT/mTOR signaling pathway and downregulating the Wnt/β-catenin pathway activity, combined with fulvestrant, significantly improves PFS.
	Ipatasertib	ATP competitive AKT inhibitor	A Phase Ib study explored the use of ipatasertib combined with fulvestrant and palbociclib in HR/HER2-negative metastatic breast cancer, and a Phase II clinical study evaluated this drug combined with paclitaxel for treating metastatic triple-negative breast cancer.	When inhibiting AKT, side effects like gastrointestinal reactions are quite obvious.
	Everolimus	mTOR inhibitor	It has been approved for use of combined exemestane in HR+/HER2+ advanced breast cancer that has progressed after previous treatment with aromatase inhibitors.	Lower bone turnover markers, control the progression of bone metastases, and extend PFS.

### 3.2 TGF-β Signaling Pathway

#### 3.2.1 Overview of the TGF-β Signaling Pathway

TGF-β is an evolutionarily highly conserved family of secreted polypeptide factors, comprising 33 human encoding genes that produce cytokines secreted as homodimers or heterodimers [55]. Under normal physiological conditions, TGF-β regulates numerous physiological processes, including early embryonic development, organogenesis, immune regulation, tissue repair, and adult homeostasis. Under pathological conditions, overexpression of TGF-β leads to extensive metabolic disorders and dysfunction, promotes EMT, ECM deposition, and cancer-associated fibroblasts (CAF) formation, thereby contributing to immune dysfunction, fibrotic diseases, and cance [56,57,58]. TGF-β is primarily secreted and stored in the ECM as a latent complex, and only activated TGF-β can bind to the TGF-βR complex to exert its biological functions [59].

Transforming growth factor-β proprotein (Pro-TGF-β) synthesized in the rough endoplasmic reticulum is cleaved and matured into TGF-β by furin in the Golgi apparatus. The latency-associated peptide (LAP) dimer non-covalently associates with mature TGF-β to form the small latent complex (SLC). SLC exists in two binding modes: most SLC bind to Latent TGF-β Binding Protein (LTBP) to form the large latent complex (LLC), which is anchored to ECM components such as fibronectin and fibrillin, maintaining TGF-β in an inactive state [60,61]; in regulatory T (Treg) cells, SLC binds to GARP protein for storage [62,63]. Apart from the non-proteolytic mechanisms that activate latent TGF-β, multiple hydrolases are also involved in TGF-β activation. These mainly include glycosidases (N-glycanase, neuraminidase) and proteases. The proteases are further classified into serine proteases (plasmin), aspartic proteases (cathepsin D), and zinc-dependent matrix metalloproteinases [58,64]. In the ECM, TGF-β exists in a latent form bound to LAP. Active TGF-β is liberated from the inhibitory binding of LAP via two mechanisms: proteolytic cleavage of LAP by integrin-recruited proteases, or integrin-dependent traction forces that induce conformational changes in LAP. The liberated active TGF-β subsequently binds to the TGF-β receptor complex to regulate target gene transcription, mRNA translation, as well as nuclear and cytoplasmic protein functions [65].

**Figure 2:**
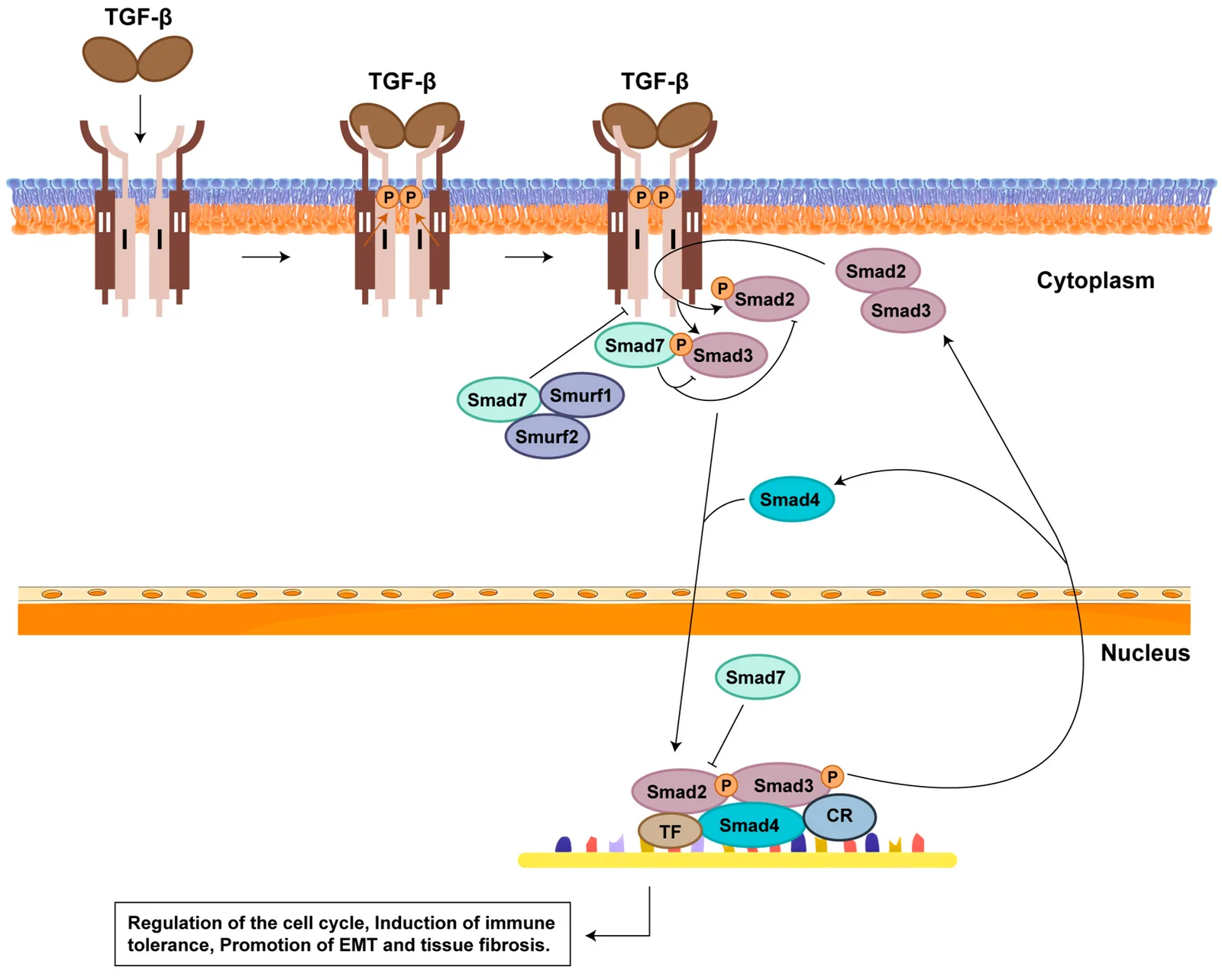
Canonical TGF-β/Smad signaling transduction pathway. Upon binding of the TGF-β ligand to the receptor, the receptor phosphorylates the receptor-regulated Smad proteins (Smad2/3). The phosphorylated R-Smads then form a complex with the common Smad protein (Smad4), which translocates into the nucleus to regulate gene expression. The inhibitory protein Smad7 can cooperate with the E3 ubiquitin ligase Smurf to mediate negative feedback degradation of the TGF-β receptor, thereby precisely modulating the activation intensity of the signaling pathway. Figure generated with Adobe Illustrator 2026 (Adobe, USA). Abb: TGF-β, transforming growth factor-β; TF, transcription factor; CR, chromatin remodeler; EMT, epithelial-mesenchymal transition.

The TGF-β pathway is divided into the canonical Smad pathway and the non-canonical non-Smad pathway, based on whether it relies on Smad family proteins as core signal transduction molecules. The Smad pathway begins with the binding of TGF-β ligand to TGF-β type receptor II (TβRII) on the cell membrane, which then recruits and phosphorylates TβRI. The activated TβRI phosphorylates receptor-regulated Smad proteins (R-Smads) (Smad2/Smad3), which further form heterotrimeric complexes with common-type Smad proteins (Co-Smad) (Smad4). This complex then translocates from the cytoplasm into the nucleus, where it binds to DNA-binding transcription factors and co-regulators, thereby regulating the transcription of numerous target genes involved in various cellular functions, including cell growth, differentiation, apoptosis, EMT, and pro-fibrotic processes. Downstream factors of Smad signaling include both positive and negative regulators: Smad2 and Smad3 are positive regulators that promote TGF-β signaling in tissue fibrosis and tumorigenesis. In contrast, Smad6 and Smad7 are considered inhibitory Smad proteins (I-Smads) that ameliorate TGF-β-mediated tissue fibrosis and tumorigenesis [66,67,68,69] (Fig. 2).

In addition to transducing signals via Smad proteins, TGF-β exerts diverse biological functions through non-Smad pathways. Ligand-activated receptors recruit and activate a variety of downstream signaling molecules through post-translational modifications such as phosphorylation, acetylation, succinylation and ubiquitination, or direct protein-protein interactions. These Smad-independent intracellular signaling cascades are collectively termed non-Smad pathways, mainly including the extracellular signal-regulated kinase 1/2 (ERK1/2) pathway, Jun N-terminal kinase (JNK) pathway, p38 mitogen-activated protein kinase (MAPK) pathway, PI3K/AKT pathway, Rho-like GTPases pathways. Non-Smad pathways are not merely auxiliary regulators of Smad signaling. They can synergize with or antagonize Smad pathways to fine-tune cellular responses to TGF-β; alternatively, they independently govern critical biological processes such as cell proliferation, migration, and apoptosis [70,71].

#### 3.2.2 Role of the TGF-β Signaling Pathway in BC Bone Metastasis

##### Dual Biological Effects of the TGF-β Pathway

The TGF-β pathway exerts a dual role in cancer progression by modulating the cellular environment and cytokines; it acts as a tumor suppressor in premalignant cells and as a tumor promoter in cancer cells [72]. TGF-β induces G1 phase cell cycle arrest and apoptosis by upregulating cyclin-dependent kinase (CDK) inhibitor proteins (p15 and p21) and downregulating the proto-oncogene MYC, thereby maintaining premalignant cells in a prolonged quiescent state [73,74].

##### TGF-β Promotes Invasion and Bone Colonization of BC Cells

BC cells can secrete large amounts of TGF-β, inducing EMT and thereby acquiring enhanced abilities for migration, invasion, and anti-apoptosis, which are critical for successful bone colonization [75]. TGF-β stimulates BC cells to express the Jagged1 protein, which acts on OCs and OBs in the bone microenvironment, activating the Notch signaling pathway and promoting OBs to secrete large amounts of IL-6, accelerating OC differentiation and maturation, while also acting as a growth factor to reciprocally promote BC proliferation [76]. In addition, TGF-β can upregulate bone sialoprotein (BSP), enhancing the invasive capacity of BC cells, and can also protect BC cells from complement-mediated lysis, achieving immune evasion and creating favorable conditions for bone metastatic lesions [77].

##### TGF-β Mediates the Positive Feedback Loop of Osteolytic Bone Destruction in the Bone Microenvironment

Bone matrix serves as the body’s primary reservoir of TGF-β, with the vast majority stored in an inactive latent complex bound to bone collagen matrix. The TGF-β pathway also plays a “double-edged sword” role in bone microenvironment homeostasis. At physiological concentrations, TGF-β upregulates runt-related transcription factor 2 (Runx2) and osterix (Osx) through Smad2/3-dependent mechanisms, promoting the differentiation of mesenchymal stem cells into OBs [78], while simultaneously inhibiting RANKL expression and enhancing OPG secretion, thereby blocking OC precursor fusion and maintaining the dynamic balance between bone formation and bone resorption [79]. However, once BC cells disseminate to the bone marrow cavity, they secrete cytokines that stimulate OCs, leading to bone matrix dissolution and resorption, which releases large amounts of previously stored TGF-β in its active form. This active TGF-β then acts back on BC cells, forming a self-reinforcing positive feedback loop [80]. TGF-β, through the canonical Smad pathway, stimulates BC cells to secrete large amounts of parathyroid hormone-related protein (PTHrP), a key driver of osteolytic bone destruction, while also enhancing estrogen receptor α (ERα)-mediated transcriptional activity to further promote PTHrP production. PTHrP acts on the PTH1 receptor (PTH1R) receptor on OBs, markedly upregulating RANKL and downregulating OPG, thereby altering the RANKL/OPG ratio and rapidly inducing excessive OC differentiation and maturation, establishing a vicious cycle [81]. In addition, TGF-β stimulates BC cells to secrete cytokines such as connective tissue growth factor (CTGF), interleukin-6 (IL-6), and IL-11, which further stimulate OC activity, inhibit OB differentiation, and exacerbate bone destruction [5].

##### Non-Smad Pathways Cooperate with Canonical Smad Pathways to Facilitate BC Bone Metastasis

Non-canonical pathways (non-Smad pathways) can synergize with the Smad pathway to amplify the EMT effect, further enhancing the migratory capacity and anti-apoptotic properties of BC cells. TGF-β activates PI3K and initiates the AKT cascade, which inhibits the mitochondrial apoptosis pathway in BC cells, promotes their survival in the bone microenvironment, and simultaneously stimulates vascular endothelial growth factor (VEGF) secretion to induce neovascularization in bone metastases, providing a nutritional blood supply. The p38/MAPK and JNK pathways can cooperate with Smad proteins to enhance the transcription of PTHrP and IL-6, accelerating OC differentiation, and regulating the expression of integrin α5β1, which facilitates the specific adhesion of BC cells to the bone endosteum and enhances bone homing efficiency. The epigenetic regulator EZH2 can synergize with the TGF-β pathway to activate focal adhesion kinase (FAK), and FAK reciprocally phosphorylates TGF-β receptors, further enhancing Smad pathway activity and accelerating the progression of BC bone metastasis [82,83].

##### TGF-β Pathway Mediates Immunosuppression and Immune Evasion in Bone Microenvironment

The activated TGF-β signaling pathway is critical for establishing an immunosuppressive barrier within the bone microenvironment, creating conditions for immune evasion by BC cells. On one hand, TGF-β induces the proliferation of regulatory T (Treg) cells in the bone marrow, suppressing the tumor-killing functions of cytotoxic T lymphocytes (CTLs) and natural killer (NK) cells. In a 2013 study, Hanks et al. found that in BC, the loss of TGF-βRIII and its shed extracellular domain (sTGF-βRIII) significantly enhances TGF-β signaling within dendritic cells (DCs), thereby inducing Treg cell infiltration and immunosuppression, accelerating BC progression and metastasis [84]. On the other hand, the TGF-β pathway promotes the secretion of chemokines by BC and stromal cells, recruiting tumor-associated macrophages (TAMs) and polarizing them toward the M2 pro-tumor phenotype. M2-type TAMs further release pro-tumor and pro-osteoclastogenic cytokines while suppressing antigen-presenting capacity. Additionally, the TGF-β pathway downregulates MHC molecule expression on BC cells, reducing immune cell recognition efficiency and enabling bone metastatic lesions to evade immune surveillance. The TGF-β signaling pathway can also transactivate epidermal growth factor receptor (EGFR) via Smad3 and the ERK/specificity protein 1 (Sp1) signaling pathway, subsequently continuously amplifying downstream signals through EGFR/PI3K/AKT and EGFR/MAPK/ERK positive feedback loops, promoting EMT, MMP-9 secretion, and cytoskeletal remodeling, thereby enhancing BC cell proliferation, migration, and invasion. This provides a theoretical basis for combined targeting strategies against TGF-β and EGFR [85].

#### 3.2.3 Therapeutic Strategies Targeting the TGF-β Pathway in BC Bone Metastasis

The TGF-β pathway sustains the osteolysis–TGF-β release–re-osteolysis vicious cycle that maintains BC bone metastasis. Current therapeutic strategies have established a ligand–receptor–downstream three-tier targeting system, which is divided into three categories: ligand-neutralizing antibodies, receptor kinase small-molecule inhibitors, and intracellular Smad signaling modulation. These are combined with conventional treatments such as immunotherapy, bone protection, chemotherapy, and targeted therapy, supplemented by novel approaches including bone-targeted delivery, natural product modulation, and gene editing.

##### TGF-β Ligand-Neutralizing Agents

Ligand-neutralizing agents can directly capture free activated TGF-β, cutting off the extracellular signal source, and are divided into two categories: broad-spectrum neutralizing antibodies and receptor Fc fusion proteins. The classic broad-spectrum neutralizing antibody is 1D11, which can simultaneously bind to all TGF-β isoforms (I/II/III), directly blocking TGF-β-induced transcription of PTHrP and GLI Family Zinc Finger 2 (GLI2), downregulating RANKL expression, upregulating OPG, inhibiting excessive OC differentiation, while also reversing EMT and reducing the invasive capacity of BC cells. 1D11 can reduce the bone metastatic burden by 70%–80%, significantly increase bone mineral density and trabecular thickness, and substantially reduce osteolytic bone defects [86]. The first-generation humanized antibody derived from 1D11, Fresolimumab (GC1008), has completed Phase II clinical studies in advanced triple-negative BC, demonstrating significant reduction in peripheral blood TGF-β levels and relief of immunosuppression, but its monotherapy objective response rate (ORR) is relatively low, with susceptibility to skin toxicity and cardiovascular adverse effects [87]. The second-generation humanized antibody SAR439459 exhibits higher plasma TGF-β inhibition rates and remodels the bone immune microenvironment, but carries safety concerns including bleeding and cutaneous squamous cell carcinoma. In recent innovative development, the bone-targeting trap protein D10-Fc-T2m is a receptor Fc fusion protein that utilizes the extracellular domain of TβRII to specifically bind and neutralize TGF-β, with its Fc fragment prolonging half-life and enhancing protein stability; and coupled with the bone-targeting peptide D10 composed of polyaspartic acid, which specifically binds to bone hydroxyapatite, achieving enrichment at bone lesions [88].

##### Small-Molecule Inhibitors of TGF-β Receptor Kinases

Receptor kinase small-molecule inhibitors target the serine/threonine kinase domain of TGF-βR on the cell membrane, preventing receptor phosphorylation and downstream Smad pathway activation. This represents a relatively mature small-molecule direction for clinical translation and is divided into TGF-βR I inhibitors and TGF-βR II inhibitors. Galunisertib (LY2157299) is a classic dual-target ALK5 inhibitor that blocks TGF-βR I phosphorylation while mildly inhibiting TGF-βR II, suppressing Smad2/3 phosphorylation and thereby blocking the Smad pathway. This inhibits BC cell secretion of PTHrP, IL-6, and CTGF, downregulates RANKL and upregulates OPG in OBs, corrects the RANKL/OPG ratio imbalance, significantly reduces OC activity, and attenuates osteolytic effects, markedly delaying the onset of SREs such as pathological fractures, refractory bone pain, spinal cord compression, and hypercalcemia [86]. TGF-βR II inhibitors specifically block receptor dimerization and modulate TGF-β-induced cell growth effects [89].

##### Intracellular Regulation of Smad Signaling

Intracellular Smad signaling modulators target the pathway inhibitory protein Smad7 as a core target, upregulating Smad7 expression to positively inhibit the pathway and reinforce endogenous negative feedback. These are divided into Smad7 gene overexpression/stabilization and Smurf ubiquitin ligase inhibitors. Lentiviral vector-mediated stable overexpression of Smad7 in tumor cells and bone marrow stromal cells competitively binds to TGF-βRI, recruits Smurf ubiquitin ligase to degrade the receptor, and simultaneously inhibits Smad2/3 nuclear translocation, completely reversing TGF-β-induced RANKL upregulation and OC activation, with a significant reduction in osteolytic lesions in mouse models [90,91]. The deubiquitinase OTUD1 specifically removes ubiquitin chains from Smad7, preventing its proteasome-mediated degradation by Smurf1/2, stabilizing total intracellular Smad protein levels, reducing TGF-β pathway signaling, and blocking the osteolytic vicious cycle [92]. Smurf ubiquitin ligase inhibitors, such as bicyclic phage display peptides, block the binding of Smurf2 to Smad7, reducing Smad7 degradation, downregulating intracellular pSmad2/3, inhibiting TGF-β-induced EMT and PTHrP, and indirectly enhancing pathway negative regulation. However, these peptides have poor stability and are susceptible to proteolysis, and remain in preclinical developmen [93]. The treatment regimens targeting the TGF-β signaling pathway of breast cancer bone metastasis are listed in Table 2.

### 3.3 The RANK/RANKL/OPG Signaling Pathway

#### 3.3.1 Overview of the RANK/RANKL/OPG Signaling Pathway

**Figure 3:**
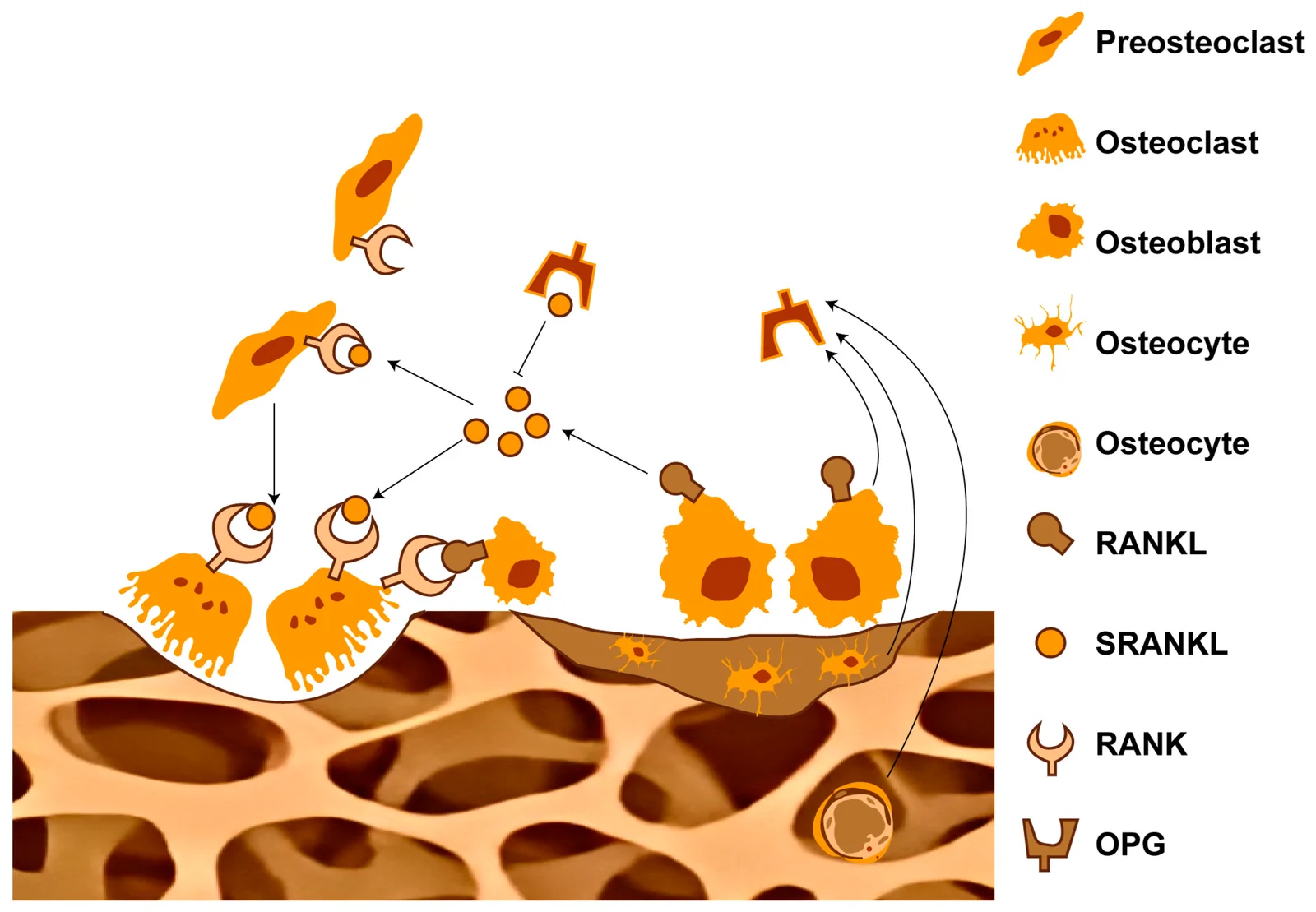
RANKL/RANK/OPG signaling mediates osteoclast (OC) activation and osteolytic bone destruction in the bone microenvironment. Osteoblasts (OB) secrete receptor activator of nuclear factor-κB ligand (RANKL), which binds to receptor activator of NF-κB (RANK) receptors on the surface of OC precursor cells, inducing their differentiation into mature OCs and thereby promoting bone resorption. Meanwhile, OBs, osteocytes, and vascular endothelial cells secrete osteoclastogenesis inhibitory factor (OPG), which acts as a decoy receptor by competitively binding to RANKL, thereby inhibiting osteoclastogenesis. Figure generated with Adobe Illustrator 2026 (Adobe, USA). Abb: RANKL: Receptor Activator of Nuclear Factor-κB Ligand; sRANKL: soluble Receptor Activator of Nuclear Factor-κB Ligand; RANK: Receptor Activator of Nuclear Factor-κB; OPG: Osteoprotegerin.

The RANK/RANKL/OPG signaling pathway plays important roles in bone metabolism, mammary epithelial cell development, immune function, tumors, diabetes, and cardiovascular diseases [94]. In the late 1990s, the RANK/RANKL/OPG signaling pathway was identified as a key regulator of bone metabolism. RANKL, a member of the tumor necrosis factor-α (TNF-α) superfamily, also known as TNFSF11, ODF, OPGL, or TRANCE, is a type II transmembrane protein with a C-terminal extracellular region, mainly expressed on the cell membranes of OBs, chondrocytes, bone marrow mesenchymal stem cells, mammary epithelial cells, and activated T and B cells. RANKL exists in two forms: membrane-bound RANKL (mRANKL) and soluble RANKL (sRANKL), both possessing biological activity that promotes OC differentiation and maturation, thereby leading to bone resorption. RANK, a member of the TNF receptor superfamily, also known as TNFRSF11A, ODFR, or TANCE, is a type I transmembrane protein containing four CRDs in its extracellular N-terminus, mainly expressed on OCs, DCs, T cells, and B cells. RANK is the sole receptor for RANKL. Binding of RANKL to RANK on the surface of OCs and their precursor cells initiates downstream signal transduction, inducing precursor cell differentiation into mature OCs and inhibiting OC apoptosis. OPG, another member of the TNF receptor superfamily, also known as TNFRSF11B, is a soluble glycoprotein lacking transmembrane and cytoplasmic domains, serving as a decoy receptor secreted by OBs, osteocytes, and vascular endothelial cells. OPG contains seven functional domains (D1–D7), with the N-terminal D1–D4 domains competitively binding RANKL with high affinity, thereby blocking the interaction between RANKL and RANK, inhibiting RANK signaling, and consequently suppressing OC differentiation and maturation [95,96]. The dynamic balance among RANK, RANKL, and OPG is essential for maintaining skeletal homeostasis, and dysregulation of these three components can lead to osteoporosis or osteopetrosis. Increased RANKL expression in tumor cells may enhance bone resorption and induce bone metastasis [94,97] (Fig. 3).

When RANK binds to mRANKL or sRANKL, TRAF6 is recruited to the cytoplasmic tail of RANK. This activates the NF-κB and MAPK pathways, together with the downstream PI3K/AKT/mTOR signaling pathway, which synergistically regulate the differentiation, maturation and biological functions of OCs [98]. Among these, the NF-κB pathway promotes RANKL autocrine signaling, tumor cell proliferation, and OC activation [99], the MAPK pathway mainly upregulates AP-1 (c-Fos) transcription, significantly enhancing the differentiation potential of OC precursors [100], and the PI3K/AKT/mTOR pathway not only regulates BC cell anti-apoptosis and neovascularization in bone metastatic lesions but also sustains OC precursor survival [101]. After the upstream pathways complete the transcriptional induction of NFATc1, they synergize with calcium oscillation signals to activate the downstream calcineurin (CN)/NFATc1 pathway. Calcineurin mediates NFATc1 dephosphorylation and nuclear translocation, directly initiating the transcription of osteoclast-specific bone resorption genes such as tartrate resistant acid phosphatase (TRAP) and Cathepsin K (CTSK), ultimately reinforcing the osteolytic bone destruction effect [102].

#### 3.3.2 Role of the RANK/RANKL/OPG Signaling Pathway in BC Bone Metastasis

Bone remodeling under physiological conditions is a continuous, dynamic biological process characterized by the concurrent occurrence of osteoclast-mediated bone resorption and osteoblast-mediated bone formation. OCs create resorption cavities in bone, which are subsequently filled by OBs that synthesize new bone matrix. This process helps maintain bone strength, repair microdamage, and regulate calcium homeostasis in the body [103,104].

##### Imbalance of RANK/RANKL/OPG Mediates Positive Feedback Loop of Osteolytic Bone Destruction

BC cells secrete RANKL in an autocrine manner, or secrete PTHrP through the TGF-β/Smad pathway, further enhancing RANKL secretion by OBs, which promotes the rapid differentiation of precursor cells into mature OCs. These OCs adhere to trabecular bone and release CTSK and TRAP, degrading hydroxyapatite and the bone collagen matrix, leading to osteolysis and trabecular bone defects. Simultaneously, growth factors such as IGF, fibroblast growth factor (FGF), platelet-derived growth factor (PDGF), BMP, and TGF-β are released from the bone matrix. Insulin-like growth factor (IGF), FGF, and other growth factors promote BC cell proliferation and anti-apoptosis, resulting in the rapid expansion of micrometastatic bone lesions. TGF-β promotes BC cells to secrete PTHrP, continuously elevating the RANKL/OPG ratio and establishing a positive feedback osteolytic effect [105]. In addition, BC cells secrete a variety of cytokines, such as IL-1α, IL-6, IL-8, IL-11, TNF-α, macrophage colony-stimulating factor (M-CSF), and prostaglandin E2 (PGE2), which influence the bone microenvironment, promote RANKL expression, disrupt the balance between RANKL and OPG, favor RANKL binding to RANK, activate OC differentiation, lead to extensive bone matrix destruction, and thereby promote BC bone metastasis [106]. BC cells can also interact with OBs, causing them to produce less OPG, further enhancing the activating effect of RANKL on OCs and accelerating the bone destruction process.

##### RANK/RANKL Directly Regulates Biological Behaviors of BC Cells to Promote Bone Metastasis

The RANK/RANKL/OPG pathway not only regulates OCs but also directly acts on BC cells, modulating their cellular biological behaviors, promoting stemness maintenance, invasion, and bone homing, thereby facilitating BC bone metastasis. RANK upregulates stemness markers such as ALDH and SOX2, enriches the BC stem cell subpopulation, activates downstream anti-apoptotic pathways, and enhances the survival capacity of BC cells in the hypoxic, nutrient-deprived, and oxidative stress microenvironment of the bone marrow, collectively increasing the metastatic potential of BC and mediating resistance to chemotherapy, endocrine therapy, and anti-HER2 targeted agents, resulting in poor drug response and high relapse rates in bone metastatic lesions [107]. Furthermore, RANKL activates the NF-κB pathway in BC cells, upregulates EMT markers, reduces cell-cell adhesion, and enhances the ability of BC cells to penetrate vascular endothelium and invade the bone matrix [108]. In addition, RANKL acts as a chemokine, binding to RANK on the surface of BC cell membranes, guiding circulating tumor cells (CTCs) to migrate toward the bone microenvironment with high RANKL concentration, achieving bone-directed homing. BC subtypes with high RANK expression show a significantly increased propensity for bone metastasis [109].

##### RANK/RANKL/OPG Pathway Regulates Immunosuppression and Tumor Immune Evasion in Bone Microenvironment

Osteoimmunology is a discipline that studies the interactions between the skeletal system and the immune system. This field has revealed that the immune system, through the production of RANKL, exerts immunosuppressive effects by promoting Treg cell activation and inhibiting the antigen-presenting function of DCs [110]. Overactivation of the RANK/RANKL/OPG pathway drives the polarization of bone marrow mononuclear macrophages toward the M2-type TAM phenotype, which in turn secretes RANKL and IL-6, leading to immunosuppression and osteolytic bone destruction [111]. Therefore, RANKL serves as a critical mediator in the crosstalk between immune cells and OCs, contributing to immune evasion of bone metastatic lesions by remodeling the immunosuppressive bone marrow microenvironment [112,113].

#### 3.3.3 Therapeutic Strategies Targeting the RANK/RANKL/OPG Signaling Pathway in BC Bone Metastasis

##### Clinical Application of Mainstream Targeted Drugs

Given the important role of the RANK/RANKL/OPG signaling pathway in BC bone metastasis, targeted therapeutic strategies against this pathway have become a research hotspot. Among these, the Bone Tumor and Bone Metastasis Committee of the China Anti-Cancer Association pointed out in its 2022 “Expert Consensus on Clinical Diagnosis and Treatment of BC Bone Metastasis” that denosumab, originally known as AMG162, is a fully human monoclonal antibody that specifically targets RANKL. It is now widely used in the treatment of BC bone metastasis and other bone-related diseases. The first-line clinical monotherapy regimen for BC bone metastasis is centered on denosumab, with bisphosphonates as a classic alternative. Both agents modulate the RANKL/OPG ratio, block the downstream CN/NFATc1 pathway, and disrupt the vicious cycle of osteolytic bone destruction. Denosumab specifically neutralizes free RANKL, blocking the binding of RANKL to RANK, effectively inhibiting OC activation and maturation, thereby reducing bone resorption. Phase III clinical trials have demonstrated that, compared with zoledronic acid, denosumab more effectively reduces the risk of SREs and significantly delays their onset. Moreover, it is not metabolized by the kidneys, making it more suitable for patients with renal insufficiency or those receiving nephrotoxic chemotherapy [114,115,116,117]. The third-generation bisphosphonate zoledronic acid is deposited in the remodeling bone matrix, where it is extensively taken up and accumulated by activated OCs, thereby inhibiting the mevalonate pathway, directly inducing OC apoptosis, and indirectly downregulating RANK activity. Because it is primarily cleared by the kidneys, it is mainly used for long-term bone protection in patients with normal renal function [118,119]. Novel decoy receptor agents, such as recombinant modified OPG mutants and LGR4 extracellular domain proteins, can competitively bind RANKL and are currently in preclinical development, representing a new direction for next-generation monotherapy research [120,121].

##### Novel Combined Targeted Therapeutic Strategies

Currently, based on BC molecular subtyping, the implementation of systemic anti-tumor drug therapy in combination with pathway-targeted agents has become the standard comprehensive approach for advanced BC with bone metastases. For HR-positive patients, aromatase inhibitors combined with CDK4/6 inhibitors are selected; for HER2-positive patients, monoclonal antibodies, TKIs, or ADC drugs are used; and for triple-negative patients, chemotherapy, immune checkpoint inhibitors, or PARP inhibitors are employed, along with palliative radiotherapy for bone metastatic lesions and concurrent use of denosumab or bisphosphonates, addressing both systemic BC treatment and control of bone metastatic progression. In addition, combined targeting of upstream and downstream pathways—such as the co-administration of denosumab with TGF-β receptor inhibitors—simultaneously blocks upstream TGF-β-mediated PTHrP secretion and downstream RANKL-driven osteolytic effects, effectively interrupting signaling pathway transmission in bone metastasis [122]. The treatment regimens targeting the RANK/RANKL/OPG signaling pathway of breast cancer bone metastasis are listed in Table 2.

### 3.4 The PI3K/AKT/mTOR Signaling Pathway

#### 3.4.1 Overview of the PI3K/AKT/mTOR Signaling Pathway

##### Composition and Biological Functions of the PI3K/AKT/mTOR Pathway

The PI3K/AKT/mTOR signaling pathway is widely present and highly conserved in eukaryotic cells, with complex and close interconnections with other signaling pathways. It participates in regulating multiple biological processes including cell proliferation, differentiation, autophagy, migration, invasion, metastasis, apoptosis, cell cycle, angiogenesis, metabolism, and drug resistance. Dysfunction of this pathway, such as PI3K hyperactivation, phosphatase and tensin homolog (PTEN) mutation or inactivation, and aberrant AKT activation, can lead to disease progression and therapeutic resistance. PI3K is a class of intracellular lipid kinases, divided into three classes: PI3K class I, II, and III. Class I PI3K is further subdivided into class IA (PI3Kα, PI3Kβ, and PI3Kδ) and class IB (PI3Kγ). Class IA PI3K is a heterodimer composed of a regulatory subunit p85 and a catalytic subunit p110, while class IB PI3K is a heterodimer composed of a regulatory subunit p101 and a catalytic subunit p110. When growth factors bind to receptor tyrosine kinases (RTKs) on the cell membrane, the intracellular tyrosine residues of RTKs undergo phosphorylation. The regulatory subunit p85 of class IA PI3K binds to these via its Src homology 2 (SH2) domain, releasing the catalytic subunit p110. RAS protein in its GTP-bound activated state directly binds to the catalytic subunit p110 of class IA PI3K, assembling an active PI3K enzyme with full catalytic function, a process commonly observed in tumor cell signaling. Upon ligand activation, G protein-coupled receptors (GPCRs) dissociate the Gβγ subunits, which directly bind to the regulatory subunit p101 of class IB PI3K, a process more common in immune and hematopoietic cell signaling. Class II PI3K includes catalytic isoforms PI3KC2α, PI3KC2β, and PI3KC2γ, lacking regulatory subunits, and are monomeric catalytic isoforms involved in membrane trafficking. Class III PI3K, through binding to protein complexes composed of regulatory and catalytic subunits, plays an important role in regulating autophagy and macrophage phagocytosis [123,124,125].

As the initiating enzyme of the PI3K/AKT/mTOR pathway, class IA PI3K catalyzes the conversion of phosphatidylinositol 4,5-bisphosphate (PIP2) to phosphatidylinositol 3,4,5-trisphosphate (PIP3), which further activates AKT. AKT, also known as protein kinase B (PKB), is a serine/threonine protein kinase that includes three isoforms: AKT1, AKT2, and AKT3. PIP2 and PIP3 mediate the translocation of AKT from the cytoplasm to the cell membrane via the PH domain, leading to its activation, which in turn activates mTOR. mTOR belongs to the phosphatidylinositol kinase-related kinase (PIKK) protein superfamily, whose members all possess a catalytic domain similar to that of the lipid kinase PI3K but function as serine/threonine protein kinases [126]. As a catalytic subunit, mTOR assembles with different proteins to form two multi-protein complexes, mTORC1 and mTORC2. mTORC1 is highly sensitive to rapamycin and regulates biological processes such as protein and lipid synthesis and autophagy. mTORC2 is insensitive to rapamycin and regulates cell survival, cytoskeletal reorganization, and metabolic balance by phosphorylating members of the AGC kinase family, such as AKT, PKCα, and serum and glucocorticoid-regulated kinase 1 (SGK1) [127,128]. PTEN is a classic tumor suppressor protein and serves as a key negative regulator of this signaling pathway. It possesses phospholipid phosphatase activity that dephosphorylates the second messenger PIP3 generated by PI3K, converting it back to PIP2, thereby terminating PI3K-mediated downstream pro-survival signal transduction [129].

##### Classical Activation Mechanism of the PI3K/AKT/mTOR Signaling Pathway

**Figure 4:**
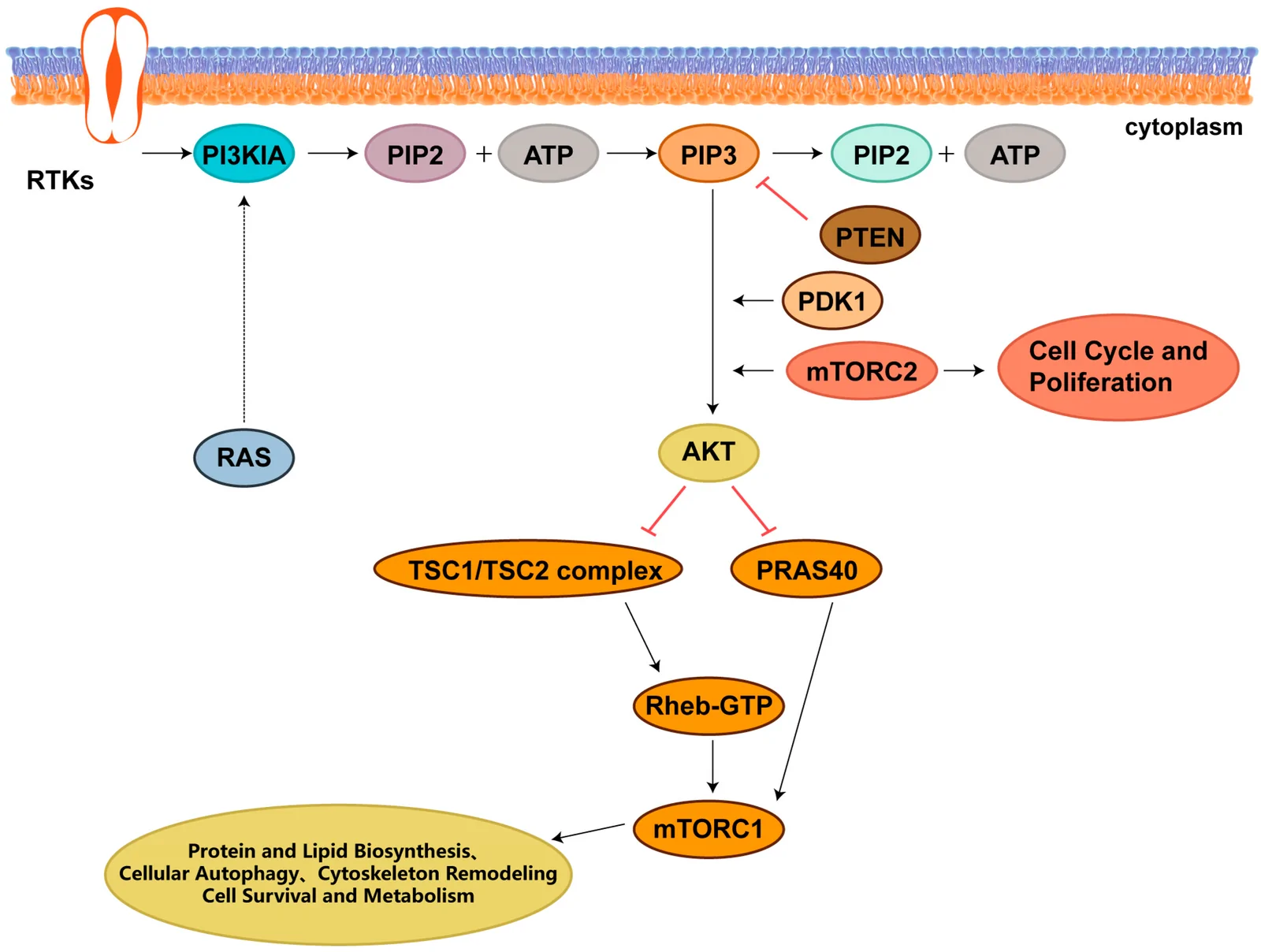
Schematic diagram of the canonical activation of the PI3K/AKT/mTOR signaling pathway. Upon activation of RTKs by growth factors, PI3KIA is activated, which catalyzes the production of PIP3. PIP3 subsequently recruits AKT and PDK1 to the cell membrane. PDK1 phosphorylates AKT, and mTORC2 further phosphorylates AKT, achieving its full activation. Activated AKT inhibits the TSC1/TSC2, relieving its inhibitory effect on Rheb-GTP, which in turn activates mTORC1. Meanwhile, AKT can phosphorylate and inhibit PRAS40, relieving its blocking effect on mTORC1. PTEN dephosphorylates PIP3, thereby exerting a negative regulatory role on the entire pathway. Figure generated with Adobe Illustrator 2026 (Adobe, USA).

When ligands such as EGF and IGF-1 bind to RTKs on the cell membrane, the intracellular tyrosine residues of the RTKs undergo autophosphorylation. Class IA PI3K binds to these phosphorylated tyrosine sites, thereby being recruited to the cell membrane and undergoing conformational activation. Activated class IA PI3K catalyzes the phosphorylation of PIP2 to generate the second messenger PIP3. PIP3 simultaneously anchors AKT and 3-phosphoinositide-dependent protein kinase 1 (PDK1) via their PH domains and recruits them to the cell membrane. PDK1 first phosphorylates AKT at Thr308, followed by mTORC2 phosphorylation of AKT at Ser473, achieving full AKT activation [129]. Activated AKT phosphorylates and inhibits the tuberous sclerosis complex 1/2 (TSC1/TSC2), relieving its inhibitory effect on Rheb-GTP, allowing active Rheb-GTP to accumulate and allosterically activate mTORC1 [130]. In addition, activated AKT phosphorylates proline-rich AKT substrate of 40 kDa (PRAS40), relieving its inhibition of mTORC1 [131]. mTORC1 directly phosphorylates eIF4E-binding protein (4E-BP1), releasing eIF4E to initiate cap-dependent mRNA translation, and also activates ribosomal protein S6 kinase beta-1 (S6K1) to promote ribosomal synthesis, enhancing tumor cell nutrient uptake and utilization efficiency, ultimately driving tumor cell metabolic reprogramming and malignant biological behaviors [132,133,134,135] (Fig. 4).

#### 3.4.2 The Role of the PI3K/AKT/mTOR Signaling Pathway in BC Bone Metastasis

##### Bidirectional Regulation of Bone Remodeling by the PI3K/AKT/mTOR Pathway

The PI3K/AKT/mTOR signaling pathway plays a dual regulatory role in bone remodeling. On one hand, it is a key driver of OB proliferation, differentiation, and bone matrix synthesis. Among these ligands, IGF-1 activates RTKs on the surface of preosteoblasts. This recruits PI3K to the cell membrane and triggers its activation; activated PI3K converts PIP2 into PIP3, which in turn induces AKT phosphorylation and subsequent mTORC1 activation. This cascade upregulates Runx2 and Osx expression, accelerating robust secretion of type I collagen, matrix deposition, and the formation of mineralized nodules. In addition, although BMP-2 transduces signals via its specific serine/threonine kinase receptors (BMPRs), its downstream signaling can also cross-activate the PI3K/AKT pathway through non-Smad cascades to synergistically promote osteogenic differentiation [136,137]. On the other hand, this pathway is a critical driver of OC proliferation and differentiation. Phosphorylated AKT inhibits GSK3β, stabilizing the NFATc1 protein, thereby upregulating DCSTAMP and CTSK and promoting the maturation of multinucleated OCs [138]. Notably, it exhibits a dose-dependent, bidirectional regulatory effect on bone metabolism: moderate activation under physiological conditions promotes osteogenesis, whereas pathological, sustained overactivation leads to excessive RANKL secretion by OBs, indirectly accelerating OC differentiation and bone resorption activity, thereby promoting osteoclastogenesis and disrupting the bone metabolic balance [139].

##### PI3K/AKT/mTOR Pathway Mediates Osteolytic Bone Metastasis of BC

The PI3K/AKT/mTOR signaling pathway is a key regulator of BC bone metastasis, and together with the RANK/RANKL/OPG and TGF-β pathways, it constitutes a complex regulatory network governing bone metastasis [5]. Within BC cells, the PI3K/AKT/mTOR pathway directly promotes cell proliferation, migration, and invasion. mTORC1 promotes ribosomal synthesis and protein translation, providing necessary biosynthetic materials for BC cell growth, thereby driving proliferation. In addition, mTORC1 influences BC cell survival and adaptability by regulating processes such as autophagy [128]. Activated AKT phosphorylates substrates including B-cell lymphoma-2 gene-related promoter (BAD), FOXO, and GSK3, inhibiting BC cell apoptosis while promoting protein synthesis, immune evasion, and angiogenesis. Furthermore, this pathway forms a complex cross-regulatory network with pathways such as Notch, insulin-like growth factor 1 receptor (IGF-1R), and TGF-β, playing a critical role in maintaining BCSC properties [140]. At the bone microenvironment level, the PI3K/AKT/mTOR pathway significantly promotes BC cell secretion of PTHrP and IL-6, upregulates RANKL expression, and downregulates OPG in OBs. This aberrantly activates the RANK/RANKL/OPG pathway, enhances downstream CN/NFATc1 signaling, promotes OC differentiation and bone resorption, and induces osteolytic bone destruction. This releases large amounts of TGF-β and IGF-1, which in turn, through the RTK and non-canonical TGF-β pathways, respectively, reciprocally activate the PI3K/AKT/mTOR pathway in BC cells, forming a positive feedback vicious cycle [105,141,142,143].

#### 3.4.3 Therapeutic Strategies Targeting the PI3K/AKT/mTOR Signaling Pathway in BC Bone Metastasis

Given that PTEN loss or inactivating mutations, AKT gene amplification or activating mutations, and PIK3CA mutations all lead to sustained hyperactivation of the PI3K/AKT/mTOR signaling pathway, thereby driving BC bone metastasis, inhibitors targeting this pathway have become a research hotspot in BC therapy. Currently, a variety of PI3K, AKT, and mTOR inhibitors have demonstrated favorable anti-tumor efficacy.

##### Clinical Application of PI3K Inhibitors

PI3K inhibitors represent the most well-established class of targeted therapeutics for this pathway. For patients with PIK3CA-mutated, HR+/HER2− advanced or metastatic BC, the selective PI3Kα inhibitors alpelisib and inavolisib are often used in combination with fulvestrant and the CDK4/6 inhibitor palbociclib. By blocking PI3K activity, they inhibit BC cell secretion of PTHrP and IL-6, reduce RANKL overexpression at the source, indirectly attenuate OC activation potential, and prolong overall survival [144,145,146].

##### Research Progress of AKT Inhibitors

In recent years, AKT inhibitors have also made significant progress. Capivasertib occupies the ATP-binding pocket of the AKT kinase domain, hindering ATP binding and energy supply, blocking AKT phosphorylation, and simultaneously inhibiting all three isoforms AKT1, AKT2, and AKT3, thereby blocking downstream signal transduction. GSK-3β activity is restored, stabilizing the β-catenin degradation complex and downregulating Wnt/β-catenin pathway activity, inhibiting BC cell proliferation and EMT. Phase III clinical trials have confirmed that this drug, in combination with fulvestrant, significantly improves progression-free survival (PFS) in patients with HR+/HER2− advanced BC who have experienced recurrence or progression during or after aromatase inhibitor-based endocrine therapy, making it the first selective AKT inhibitor approved globally [147]. Ipatasertib is another highly selective ATP-competitive AKT inhibitor. A Phase Ib clinical study explored ipatasertib in combination with fulvestrant and the CDK4/6 inhibitor palbociclib in patients with HR+/HER2− metastatic BC, achieving a median PFS of 5.5 months, a median overall survival (OS) of 24.5 months, and a clinical benefit rate (CBR) of 48% [148]. A Phase II clinical study evaluated ipatasertib in combination with paclitaxel for metastatic triple-negative BC patients who had not received prior first-line chemotherapy for advanced disease, showing improved median PFS but with significant gastrointestinal adverse effects [149]. A meta-analysis indicated that AKT inhibitors hold promising prospects for treating advanced or metastatic BC with PIK3CA/AKT1/PTEN mutations, significantly improving OS and PFS [150].

##### Therapeutic Value of mTOR Inhibitors in BC Bone Metastasis

mTOR inhibitors are increasingly valued in the treatment of BC bone metastasis. Everolimus, by inhibiting mTOR activity, affects BC cell metabolism and growth, and has shown inhibitory effects on BC bone metastasis [151,152]. In a Phase III clinical trial, everolimus in combination with exemestane was used in HR+/HER2− advanced BC patients who had progressed after prior aromatase inhibitor therapy. Compared with exemestane alone, the combination significantly prolonged median PFS, reduced bone turnover marker levels, effectively controlled bone metastatic lesion progression, and alleviated bone pain [153].

##### Therapeutic Effects of Natural Small-Molecule Compounds on BC Bone Metastasis

In addition to synthetic targeted inhibitors, natural small-molecule compounds also possess the potential to suppress BC bone metastasis. Ononin represents a candidate agent against BC bone metastasis. By targeted inhibition of MAPK pathway activation, ononin exerts dual effects: on the one hand, it inhibits the proliferation, migration and invasion of triple-negative BC cells and reduces the secretion of osteolytic factors by BC cells; on the other hand, it blocks RANKL-mediated OC maturation, downregulates OC functional genes such as CTSK, and alleviates osteolytic bone lesions. It exhibits potent anti-bone-metastasis activity in nude mouse models of BC bone metastasis without obvious organ toxicity [154]. The treatment regimens targeting the PI3K/AKT/mTOR signaling pathway of breast cancer bone metastasis are listed in Table 2.

## 4 Cross-Regulation of Key Signaling Pathways Driving BC Bone Metastasis

During the progression of breast cancer bone metastasis, the four major signaling pathways—Wnt/β-catenin, TGF-β, RANK/RANKL/OPG, and PI3K/AKT/mTOR—do not function independently. Instead, they are interconnected and cross-regulate one another to collectively facilitate breast cancer cell proliferation, bone colonization, and remodeling of the bone immune microenvironment [12]. With the RANK/RANKL/OPG axis serving as the terminal effector pathway governing bone metabolism, the other three pathways exert synergistic activating effects and form upstream and downstream cascades. Together, they sustain the stemness and immune evasion capacity of breast cancer cells, ultimately generating a self-amplifying vicious cycle of osteolytic bone destruction.

### 4.1 Positive Feedback Regulation among the PI3K/AKT/mTOR Pathway, Wnt/β-Catenin Pathway, and RANK/RANKL/OPG Pathway

Upon binding of Wnt ligands to cell membrane receptors, the Wnt/β-catenin signaling pathway is activated, and nuclear β-catenin transcriptionally upregulates insulin receptor substrate 1 (IRS-1), enhancing the sensitivity of BC cells to IGF-1, which in turn recruits class IA PI3K and activates the PI3K/AKT/mTOR pathway [155]. GSK3β is a key kinase of the β-catenin degradation complex; activated AKT phosphorylates and inhibits GSK3β, causing β-catenin to escape ubiquitination and degradation, allowing it to accumulate in the nucleus to bind to the TCF/LEF transcriptional complex, upregulating target genes such as Snail, Twist, Runx2, and Cyclin D1, thereby forming a positive feedback signal amplification loop [156,157]. At the same time, activated AKT can directly phosphorylate β-catenin at Ser552, further enhancing its nuclear transcriptional activity and exerting an immediate activation effect [158]. Under sustained activation of BC cells, the PI3K pathway directly stimulates BC cells to secrete large amounts of PTHrP, while the Wnt pathway indirectly promotes PTHrP secretion through GLI2, thereby upregulating RANKL expression in OBs and inducing osteolytic bone destruction [159,160].

### 4.2 Deep Crosstalk among the PI3K/AKT/mTOR Pathway, TGF-β Pathway, and RANK/RANKL/OPG Pathway

After TGF-β binds to TβRI/II, it recruits the ubiquitin ligase TRAF6, which undergoes K63-linked ubiquitination, directly binds to and activates the p85 regulatory subunit of PI3K, bypassing Smad proteins and initiating the PI3K/AKT/mTOR pathway, upregulating hypoxia-inducible factor 1-alpha (HIF-1α) and c-Myc, and enhancing the anti-apoptotic and angiogenic capacity of BC cells [71]. At the same time, activated AKT can phosphorylate Smad3, promoting its nuclear translocation and the formation of a transcriptional complex with β-catenin; mTORC1, through the S6K/4E-BP1 translational regulatory axis, enhances the translation efficiency of Smad3 downstream proteins, reinforcing canonical Smad signaling and forming a positive feedback loop [161,162,163]. Overactivation of the PI3K/AKT/mTOR pathway promotes osteoclast-mediated degradation of bone matrix, releasing large amounts of TGF-β, which again activates the PI3K pathway in tumor cells, further stimulating BC cells and osteogenic stromal cells to secrete PTHrP and IL-6. These act on OBs, promoting RANKL expression and suppressing OPG secretion, thereby activating OCs and enhancing bone resorption, ultimately generating an irreversible cascade amplification effect [109,164].

### 4.3 Cross-Regulation between the Wnt/β-Catenin Pathway and the TGF-β Pathway

TGF-β, through the Smad3-dependent pathway, upregulates the expression of Wnt3 ligand in BC cells. The increased secretion of Wnt3 ligand further activates the Wnt/β-catenin pathway, forming a positive cross-regulatory loop between the Wnt/β-catenin and TGF-β pathways, which efficiently induces EMT, synergistically enhances the invasive capacity of BC cells, and promotes BC bone metastasis.

## 5 Research Hotspots and Trends

### 5.1 Research Hotspots on the Wnt/β-Catenin Signaling Pathway in BC

Huang et al. proposed and validated an innovative combined cell therapy for the treatment of triple-negative BC in 2026, whose novelty integrates a Wnt/β-catenin signaling pathway inhibitor, death receptor-mediated apoptosis, and immune cell-based targeted delivery vectors into a single therapeutic modality. This approach holds potential for tumor targeting, direct cytotoxicity, and remodeling of the tumor microenvironment, representing a translational research trend from basic science to clinical medicine [165]. Zhang et al. discovered in 2026 that MIR4435-2HG promotes BC progression through the miR-205-5p/UBE2N axis and exosome-mediated M2 polarization of macrophages, revealing that a long non-coding RNA (lncRNA) both directly regulates tumor cells and remodels the tumor immune microenvironment via the downstream Wnt/β-catenin signaling pathway, collectively driving BC malignancy. MIR4435-2HG, its downstream target UBE2N, or the exosome secretion process have all emerged as potential intervention targets, representing a frontier trend in current cancer research [166].

### 5.2 Research Hotspots on the TGF-β Signaling Pathway in BC

El Samarji et al. revealed in 2026 that in senescent triple-negative breast cancer (TNBC) cells, the TGF-β and Wnt/β-catenin signaling pathways form a self-reinforcing positive feedback loop. This loop drives the cells to exhibit a pro-fibrotic and stem cell-like malignant phenotype, conferring enhanced self-renewal capacity, drug resistance, and tumor-initiating potential [167]. Dharwal et al. demonstrated in 2026 that in TNBC lacking estrogen receptors, the anti-estrogen drug tamoxifen initially induces oxidative stress, DNA damage, and apoptosis through non-classical, receptor-independent pathways, thereby inhibiting growth. However, long-term use activates the TGF-β signaling pathway, inducing pro-metastatic and pro-survival adaptive changes, including the acquisition of EMT, enhanced stem cell-like properties, and pro-fibrotic characteristics, which may ultimately lead to treatment failure and an increased risk of metastasis and recurrence [168].

### 5.3 Research Hotspots on the RANK/RANKL/OPG Signaling Pathway in BC

Elgohary et al. revealed in 2025 that BC stem cells upregulate the RANK/RANKL/OPG pathway, leading to an abnormally elevated RANKL/OPG ratio, enhanced bone resorption, and driving osteolytic bone metastasis [169]. Yuan et al. demonstrated in 2025 that BC cells secrete calcium-dependent annexin A2 (ANXA2), which acts as a signal amplifier by activating the STAT3 signaling pathway to enhance RANKL-induced OC differentiation, thereby exacerbating osteolytic bone metastasis [170].

### 5.4 Research Hotspots on the PI3K/AKT/mTOR Signaling Pathway in BC

Xu et al. (2026) aimed to address the issue of immune evasion in BC cells mediated by high PD-L1 expression and proposed and validated an innovative strategy to enhance immunotherapy efficacy by targeting tumor stemness. A novel therapeutic peptide targeting the stemness transcription factor SALL4 (PEN-FFW) was shown to inhibit the PI3K/AKT/mTOR signaling pathway, thereby downregulating tumor PD-L1 and enhancing the cytotoxic killing capacity of CD8^+^ T cells, achieving a dual effect of suppressing cancer stem cell properties and reversing the immunosuppressive microenvironment [171]. Yin et al. (2026) aimed to address the clinical challenge of primary or acquired resistance to PD-1/PD-L1 immune checkpoint inhibitors in BC and designed an intelligent targeted delivery system. BC stem cell-derived exosomes were modified with the TMTP1 tumor-targeting peptide for specific recognition and enrichment in BC cells and loaded with the anti-tumor natural compound salidroside. By inhibiting the hyperactive PI3K/AKT/mTOR signaling pathway in BC cells, this system reversed the immunosuppressive microenvironment [172].

## 6 Conclusion

The four major signaling pathways—Wnt/β-catenin, TGF-β, RANK/RANKL/OPG, and PI3K/AKT/mTOR—synergistically participate in driving BC bone metastasis. These pathways have preliminarily elucidated, at the molecular level, the entire process by which BC cells progress from invasion and migration to bone colonization, and have provided a theoretical foundation for targeted intervention. Although significant progress has been made in the study of signaling pathways in BC bone metastasis, our understanding of these pathways remains incomplete. Outstanding issues—such as how pathways cooperate or antagonize each other, how BC cells remodel the bone immune microenvironment, and how to overcome targeted therapy resistance—represent major obstacles between basic research and clinical translation that urgently need to be addressed. In-depth investigation into the molecular mechanisms of BC bone metastasis is imperative. Future research should not remain confined to single signaling pathways but should focus intensively on cross-regulatory networks among pathways, the bone immune microenvironment, and drug development. Leveraging cutting-edge technologies such as multi-omics integration and single-cell sequencing, researchers should identify key nodal molecules and feedback regulatory mechanisms within these pathways, thereby facilitating the translation of basic research into clinical applications. This holds promise for improving current therapeutic approaches, leading to the development of drugs with better efficacy, lower drug resistance, and fewer side effects, ultimately enabling more effective control of BC bone metastasis, improving patients’ quality of life, and prolonging their distant metastasis-free survival (DMFS), disease-free survival (DFS), progression-free survival (PFS), and overall survival (OS).

## Data Availability

Data sharing is not applicable to this article as no datasets were generated or analysed during the current study.
